# A rare case of giant teratocarcinoma of the testis with accumulation of fluid

**DOI:** 10.1016/j.eucr.2022.102057

**Published:** 2022-03-17

**Authors:** Karolina Majewska, Wiktoria Zawolik, Aleksander Targoński, Michał Tkocz

**Affiliations:** Department of Urology, Faculty of Medical Sciences, Medical University of Silesia, Katowice, Poland, Plac Medyków 1, 41-200, Sosnowiec, Poland

**Keywords:** Giant testicular tumor, Teratocarcinoma, Orchiectomy

## Abstract

Testicular cancer is one of the most curable cancers. However, the course of the disease largely depends on the clinical stage at diagnosis, and there are still cases where the tumor size is large, which makes surgical treatment challenging.

A 30-year-old man presented with painless, extremely enlarged scrotum. A CT scan revealed a tumor of the right testis of 21.5 × 15 × 18cm in size. The patient underwent a right orchiectomy and histologic examination revealed teratocarcinoma.

Suspicion of hydrocele testis should prompt meticulous differential diagnosis including malignancies. There is a strong need to increase public awareness in terms of symptoms of testicular cancer.

## Highlights

• In the presence of a giant hydrocele, palpation of the testis might prove impossible.
• Suspicion of hydrocele testis should prompt meticulous differential diagnosis.
• Ultrasound scan may underestimate the tumor size.
• In a case of giant testicular tumor inguinal - scrotal approach should be considered.
• Despite the tumor size, the patient had no metastases beyond the lymphatic system.

## 1 Introduction

Hydroceles, along with epididymal cysts, are the most common abnormalities found in scrotal ultrasonography. In the presence of a giant hydrocele, thorough palpation of the testis might prove impossible; therefore ultrasonographic examination is essential to rule out other abnormalities.

A hydrocele may coexist with testicular cancer and in up to 9%, it is the reason behind diagnostic delay.^1^ We present a case of giant enlargement of the scrotum due to a hydrocele caused by a huge mixed germ cell tumor composed of embryonal carcinoma and teratoma.

## 2 Case report

A 30-year-old man was admitted to the Urology Department in February 2020 for diagnosis and treatment of painless scrotal swelling of an undetermined cause. He first noticed the swelling three weeks before admission; the lesion continued to grow causing walking difficulties. The patient had no history of urological disease. On examination, the lesion was huge, soft and tightened the overlying skin. Palpation of the right testis was impossible; transillumination showed accumulation of clear fluid around the testicle.

**Fig. 1:**
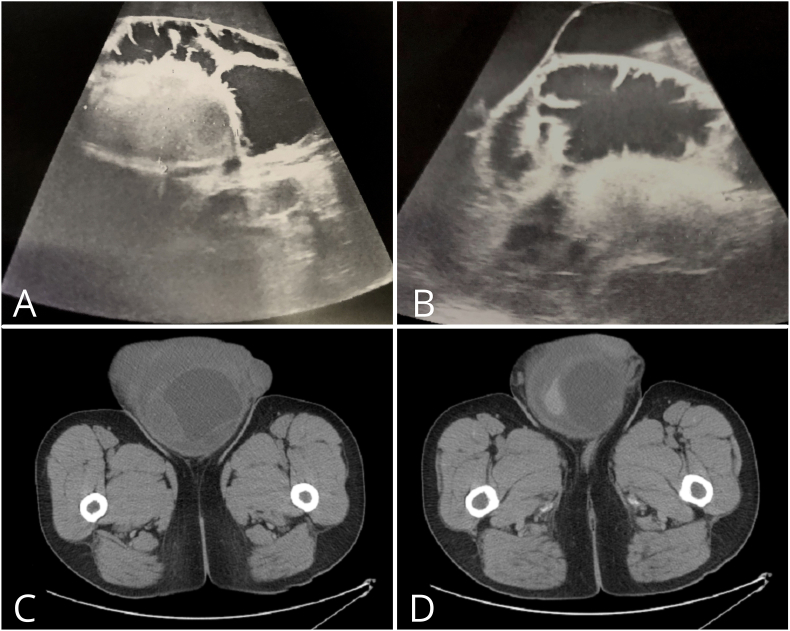
Ultrasonography of the scrotum, visible heterogeneous echotexture of the right testis and fluid collections (A). Ultrasonography of the scrotum, visible left testis and fluid collections (B). Contrast-enhanced CT scan, visible heterogeneous mixed solid and cystic mass of the right testicle (C,D).

Complete blood count performed on the day of admission revealed leukocytosis (10.52 × 10^3/μl), low hematocrit level (38.5%) and thrombocytosis (419 × 10^3/μl). Urine tests showed protein (25 mg/dl) and leukocytes (25/μl). The patient underwent an ultrasound examination (US) of the scrotum, abdomen and retroperitoneum. Two fluid collections (of 77 and 25 mm in size) were found in the scrotum, and a hydrocele was suspected (Fig. 1A and B). Due to giant scrotal enlargement, ultrasonographic assessment of the testicles proved difficult. Nevertheless, bilateral testicular enlargement, testicle asymmetry and heterogeneous echotexture of the right testis were diagnosed. Two areas indicating tumor infiltration were identified - over the right testis (40 × 23mm) and subcutaneously in the left segment of the scrotum (39 × 30 mm). Scrotal hernia was also suspected and retroperitoneal lymphadenopathy was visualized. Neck ultrasound was performed to assess a palpable mass in the thyroid gland; pathological lymph node enlargement was revealed. Considering an inconclusive ultrasound image of the right testicle, suspicion of malignancy and the presence of retroperitoneal lymphadenopathy, a contrast-enhanced computed tomography (CT) scan of the abdomen, pelvis and thorax was ordered, which confirmed a heterogeneous mixed solid and cystic mass (21.5 × 15 × 18 cm) of the right testicle (Fig. 1C and D). Retroperitoneal lymphadenopathy was also confirmed with massive conglomerate nodal mass displacing the aorta and inferior vena cava (the largest of 155 × 103 × 185 mm). Mesenteric fat infiltration, metastatic cervical lymph nodes and tracheal and right internal iliac lymph node enlargement were reported. Serum alpha – fetoprotein was 65427.7 ng/ml and human chorionic gonadotropin was 1960.29 mIU/ml.

**Fig. 2:**
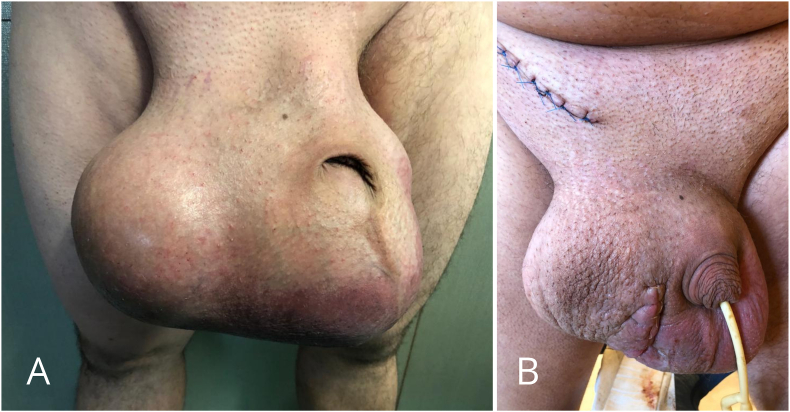
Huge enlargement of the scrotum (A). Appearance of the scrotum after right-sided orchiectomy performed with separate inguinal and scrotal incisions (B).

**Fig. 3:**
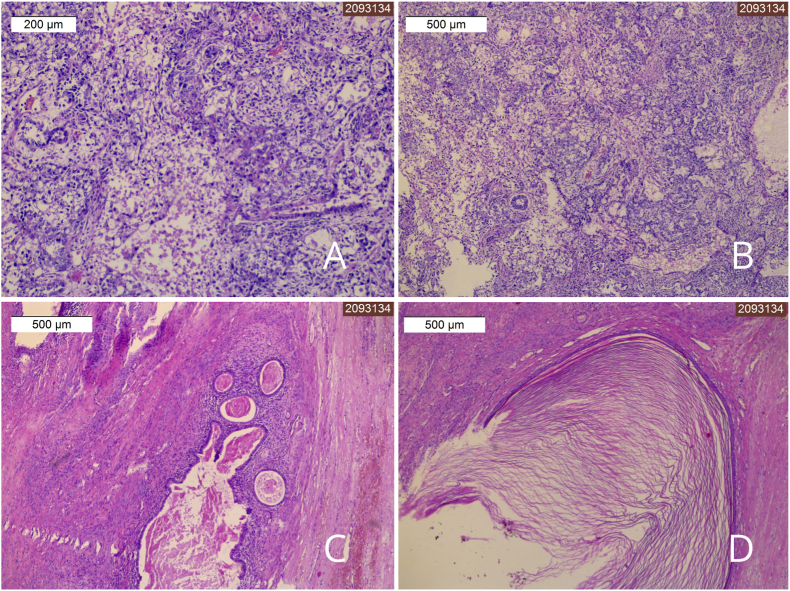
Pathology slides of the testicular tumor consisting of embryonal carcinoma and teratoma, H&E stain (A,B,C,D).

The patient was diagnosed with a right testicular tumor and scheduled for right orchiectomy. Due to the enormous size of the tumor, two separate inguinal and scrotal incisions were made (Fig. 2A and B). Firstly the incision over the inguinal canal was performed to identify and dissect the spermatic cord. Then the cord was clamped, cut and bilaterally ligated. As it was not possible to remove the enlarged testis through the inguinal canal, the separate scrotal approach was carried out. The testis was mobilized and removed via the scrotal incision. Histopathology revealed a malignant mixed germ cell tumor consisting of embryonal carcinoma and teratoma, of 14 × 13 × 12cm in size (Fig. 3A, B, 3C, 3D). The postoperative course was uneventful. The patient was discharged and referred to an oncology outpatient clinic for further treatment.

## 3 Discussion

Nowadays, due to broad access to healthcare and education, mortality rates in testicular cancer are low, however the prognosis varies significantly depending on the stage and type of cancer.

The main reasons for delay in the diagnosis of testicular cancer are insufficient knowledge and symptom neglect by the patient as well as incorrect initial diagnosis.^1^ In our case the time interval between the onset of symptoms and the diagnosis was approximately 3 weeks. Our patient, despite such a large size of the tumor, had no metastases beyond the lymphatic system indicating the disease may show different growth and spread patterns.^2^^,^^3^

Nowadays, testicular tumors of the size presented in this report are extremely rare. The average size of a testicular tumor is between 4 and 5.4 cm. Only a few giant testicular tumors have been described so far, with dimensions up to 32 × 28 cm.^2^ The hereby described tumor (14 × 13cm) was probably one of the largest testicular cancers diagnosed in Poland.

In this case, the testicular tumor was associated with the accumulation of a large amount of fluid, which hampered palpation. Initially, a hydrocele testis was suspected, which should always alert doctors to consider a neoplastic disease. Testicular ultrasound should not be delayed as it allows detection of neoplastic lesions with almost 100% sensitivity.^4^ The dimensions of our patient's right testis on the ultrasound scan were 46 × 31 mm while the entire excised specimen measured 14 × 13 × 12 cm, which indicates that the tumor size may be underestimated.

The recommended treatment of testicular cancer remains high inguinal orchiectomy, which, however, might prove inadequate to deliver a very large tumor. In such a case, inguinal - scrotal approach should be considered. Separate inguinal and scrotal incisions or one continuous incision are then made.^2^^,^^3^ Scrotal violation is controversial as it may increase the risk of metastases to regional lymph nodes or local recurrence, but it does not seem to worsen the overall prognosis.^5^ In the presented case, an inguinal incision was performed to dissect the spermatic cord and vessels, and then, due to the tumor size, the testicle was removed through a scrotal incision. Two incisions heal faster than a large one which may favorably affect the postoperative course. So far no clear recommendations have been formulated on the management of giant testicular tumors.

## 4 Conclusions

A primary diagnosis of hydrocele testis necessitates a differential diagnosis to determine the cause of the pathology, including a neoplastic process. Retroperitoneal lymphadenopathy should also prompt a physician to perform scrotal ultrasound to rule out testicular pathologies. The presented case evidences a still unsatisfactory level of society's awareness about testicular cancer. Therefore, efforts should be made to increase knowledge about this cancer, its signs and symptoms, prevention and the importance of early diagnosis.

## Consent

Written informed consent was obtained from the patient for publication of this case report and any accompanying images.

## Funding

This research did not receive any specific grant from funding agencies in the public, commercial, or not-for-profit sectors. Only the APC fee was financed under the agreement between Elsevier and Polish Consortium.

## Declaration of competing interest

Authors do not declare any conflict of interest.
